# Leveraging Artificial Intelligence and Machine Learning to Optimize Enhanced Recovery After Surgery (ERAS) Protocols

**DOI:** 10.7759/cureus.56668

**Published:** 2024-03-21

**Authors:** Zukhruf Zain, Mohammed Khaleel I. KH. Almadhoun, Lara Alsadoun, Syed Faqeer Hussain Bokhari

**Affiliations:** 1 Family Medicine, Aga Khan University Hospital, Karachi, PAK; 2 Medicine and Surgery, Mutah University, Karak, JOR; 3 Trauma and Orthopedics, Chelsea and Westminster Hospital, London, GBR; 4 Surgery, King Edward Medical University, Lahore, PAK

**Keywords:** patient risk stratification, real-time risk prediction, personalized interventions, perioperative care, ml, machine learning, ai, artificial intelligence, eras, enhanced recovery after surgery

## Abstract

Enhanced recovery after surgery (ERAS) protocols have transformed perioperative care by implementing evidence-based strategies to hasten patient recovery, decrease complications, and shorten hospital stays. However, challenges such as inconsistent adherence and the need for personalized adjustments persist, prompting exploration into innovative solutions. The emergence of artificial intelligence (AI) and machine learning (ML) offers a promising avenue for optimizing ERAS protocols. While ERAS emphasizes preoperative optimization, minimally invasive surgery (MIS), and standardized postoperative care, challenges such as adherence variability and resource constraints impede its effectiveness. AI/ML technologies offer opportunities to overcome these challenges by enabling real-time risk prediction, personalized interventions, and efficient resource allocation. AI/ML applications in ERAS extend to patient risk stratification, personalized care plans, and outcome prediction. By analyzing extensive patient datasets, AI/ML algorithms can predict individual patient risks and tailor interventions accordingly. Moreover, AI/ML facilitates proactive interventions through predictive modeling of postoperative outcomes, optimizing resource allocation, and enhancing patient care. Despite the potential benefits, integrating AI and ML into ERAS protocols faces obstacles such as data access, ethical considerations, and healthcare professional training. Overcoming these challenges requires a human-centered approach, fostering collaboration among clinicians, data scientists, and patients. Transparent communication, robust cybersecurity measures, and ethical model validation are crucial for successful integration. It is essential to ensure that AI and ML complement rather than replace human expertise, with clinicians maintaining oversight and accountability.

## Editorial

Introduction

Enhanced recovery after surgery (ERAS) protocols have revolutionized perioperative care by employing evidence-based practices to enhance patient recovery following surgical procedures [1]. These protocols encompass a multidisciplinary approach focusing on preoperative, intraoperative, and postoperative interventions to accelerate patient recovery, reduce complications, and shorten hospital stays. Their core components revolve around three pillars: preoperative optimization, minimally invasive surgery (MIS), and standardized postoperative care [2]. Preoperative optimization focuses on preparing the patient for surgery in the best possible physiological state. It includes nutritional counseling, smoking cessation, prehabilitation exercises, and psychological support, all aimed at reducing stress and improving tissue resilience. Whenever feasible, MIS techniques are prioritized to minimize tissue trauma, blood loss, and postoperative pain. This leads to faster recovery, shorter hospital stays, and an improved patient experience. Standardized postoperative care involves minimizing invasive procedures such as urinary catheters and nasogastric tubes, encouraging early mobilization and oral feeding, and implementing multimodal pain management. This promotes a faster return to bowel function, reduces complications, and improves patient comfort.

Despite their proven benefits, ERAS implementation also faces several challenges. Incomplete adherence to protocol elements across institutions and individual surgeons can limit the full potential of ERAS. This can be attributed to a lack of awareness, training, or resources. While ERAS offers a standardized approach, individual patient factors such as comorbidities, age, and body composition necessitate adjustments. Balancing standardization with individualization remains a challenge. Implementing ERAS requires dedicated personnel and infrastructure, which can be a hurdle for resource-limited healthcare systems. Artificial intelligence (AI) and machine learning (ML) hold immense potential to overcome these challenges and further optimize ERAS protocols.

Current landscape

The rise of AI and ML has revolutionized various industries, and healthcare is no exception. These powerful tools possess the ability to analyze vast amounts of data, identify patterns, and make predictions with remarkable accuracy. Healthcare providers are increasingly exploring AI and ML solutions to optimize patient care delivery, streamline clinical workflows, and enhance diagnostic accuracy. These technologies have demonstrated promising results in various medical domains, including disease prediction, treatment planning, and patient monitoring [3,4]. Their potential to transform patient care is immense, and their application in ERAS optimization holds immense promise. By incorporating real-time risk prediction and personalized interventions, AI/ML can help identify and address potential risks before they manifest as complications, leading to safer outcomes. By analyzing preoperative data, including medical history, demographics, and genetic information, AI/ML algorithms can predict the risk of developing complications in each specific patient. This allows for tailored ERAS protocols, with interventions focused on mitigating specific risks for each patient. Tailoring ERAS elements to the unique needs and vulnerabilities of each patient can promote faster and more complete recovery, reducing unnecessary interventions and improving overall well-being. Predictive models can optimize resource allocation and staffing, ensuring efficient care delivery and cost-effectiveness for healthcare systems. Predicting potential postoperative outcomes, such as length of stay or readmission risk, allows for proactive interventions and resource allocation. ERAS involves various elements, such as fluid management, pain control, and nutritional support. AI/ML can analyze individual patient data and recommend personalized adjustments to these elements, optimizing recovery and minimizing side effects [5].

Patient risk stratification

The potential of AI/ML in ERAS optimization extends beyond mere data analysis. By harnessing the power of these algorithms, we can predict individual patient's risk of complications and tailor ERAS protocols accordingly, leading to safer and more personalized recovery journeys. AI/ML algorithms can analyze vast amounts of patient data, including demographics, medical history, genetic information, and preoperative assessments. Through complex algorithms and machine learning models, they can identify patterns and relationships within this data, leading to accurate predictions of potential complications. This allows for stratification of risk as patients can be classified into low-, medium-, and high-risk groups based on their predicted complication probability. This guides the intensity and focus of ERAS interventions. Identifying patients at high risk allows for proactive measures such as targeted medication, additional monitoring, or even postponing surgery if necessary, potentially preventing complications before they occur. Knowing the predicted risk helps allocate resources efficiently, ensuring high-risk patients receive appropriate care while optimizing resource use for low-risk patients.

Personalized care plans

The journey toward recovery after surgery is a unique experience for each individual. While standardized ERAS protocols offer a solid foundation, personalization is key to optimizing outcomes and minimizing complications. AI/ML offers immense potential in this arena, allowing for dynamic adjustments to ERAS elements based on individual patient needs. Fluid balance after surgery is crucial for preventing complications such as dehydration and organ dysfunction. Traditionally, standardized fluid volumes are administered. However, AI/ML can personalize this approach by analyzing patient factors. Age, weight, body composition, medical history, and preoperative blood work are all fed into the algorithm. By considering these factors and real-time physiological data, AI/ML can predict the precise fluid volume needed for optimal recovery for each individual. As the patient progresses through recovery, the algorithm can continuously assess fluid needs and recommend adjustments, minimizing the risk of overhydration or underhydration. Effective pain management is essential for reducing suffering and promoting recovery. Analyzing genetic data and past pain experiences can predict individual sensitivity to pain medications. Based on this predicted sensitivity and risk of side effects, AI/ML can recommend the most appropriate medication and dosage for each patient, reducing the risk of over- or under-medication. Continuously analyzing pain scores, vital signs, and facial expressions using AI-powered tools can detect early signs of inadequate pain control, allowing for timely adjustments to medication or non-pharmacological interventions. Nutritional deficiencies can hamper recovery and increase the risk of complications. Preoperative blood work, dietary history, and body composition data can reveal individual nutritional needs. Based on individual needs, predicted metabolic demands, and potential risk factors such as malnutrition, AI/ML can recommend personalized calorie and nutrient intake. By tracking nutritional intake and biomarkers, the algorithm can suggest adjustments to the plan to ensure optimal nutritional support throughout recovery.

Outcome prediction

The ability to foresee potential complications and predict postoperative outcomes is paramount in optimizing patient care. Traditionally, healthcare professionals rely on clinical experience and intuition to assess risk, but this approach can be subjective and prone to error. AI/ML offers a revolutionary tool, allowing for data-driven predictions of individual patient outcomes, leading to proactive interventions and improved resource utilization. AI/ML algorithms can predict postoperative outcomes by analyzing various patient-related factors, surgical variables, and historical data. AI/ML algorithms analyze large datasets containing information on patient demographics, medical history, preoperative conditions, surgical procedures, and postoperative outcomes. By processing these data, algorithms identify patterns, correlations, and risk factors associated with different postoperative outcomes. Through feature selection techniques, AI/ML algorithms identify the most relevant variables that influence postoperative outcomes. These variables may include patient age, comorbidities, preoperative laboratory values, surgical approach, and intraoperative parameters. Once the relevant features are identified, AI/ML models are trained using supervised learning techniques. These models learn from labeled datasets where outcomes (e.g., complications and length of hospital stay) are known, enabling them to establish relationships between input variables and outcomes. After training, AI/ML models can predict postoperative outcomes for new patients based on their individual characteristics and surgical parameters. By leveraging these predictions, healthcare providers can anticipate potential complications, identify high-risk patients, and allocate resources accordingly. Early prediction of postoperative outcomes allows for proactive interventions to mitigate risks and optimize patient care. For example, if a model predicts a high risk of surgical site infection, preventive measures such as antibiotic prophylaxis or wound care protocols can be initiated preoperatively or immediately postoperatively. Predictive models enable healthcare facilities to allocate resources more efficiently by identifying patients at higher risk of complications who may require additional monitoring, intensive care unit (ICU) admission, or specialized interventions. This targeted approach optimizes resource utilization and improves patient outcomes. Through the utilization of AI/ML algorithms, healthcare providers can anticipate postoperative outcomes, intervene early to prevent complications, and allocate resources effectively, ultimately enhancing patient care and optimizing surgical outcomes.

Challenges and future directions

While the potential of AI/ML in optimizing ERAS protocols is undeniable, integrating these powerful tools into clinical practice presents several significant challenges. Sharing and accessing high-quality, diverse patient data across institutions is often hindered by privacy regulations, data ownership issues, and interoperability challenges. This can limit the development and training of robust AI models. Implementing AI/ML necessitates investments in computing power, data storage, and secured IT infrastructure. This can be particularly challenging for resource-constrained healthcare systems. Many healthcare professionals lack the necessary knowledge and skills to understand, interpret, and integrate AI/ML insights into their decision-making. Training programs and resources are crucial to bridge this gap. Implementing new technologies can evoke resistance due to concerns about job displacement, complexity, or potential ethical implications. Addressing these concerns and fostering trust through open communication is essential. AI models are trained on data, which can reflect existing societal biases. This can lead to discriminatory outcomes if not carefully addressed through diverse datasets and rigorous model validation. The "black box" nature of some AI algorithms can make it difficult to understand how they arrive at their predictions. This lack of transparency can hinder trust and raise concerns about accountability. Protecting patient data privacy and ensuring data security are paramount when using AI/ML in healthcare. Robust cybersecurity measures and clear data governance policies are essential. To navigate these challenges and unlock the full potential of AI/ML in ERAS, a human-centered approach is crucial. This involves fostering collaboration between clinicians, data scientists, and patients throughout the development and implementation process to ensure ethical and clinically relevant solutions that address patients' needs. AI/ML should not replace human expertise. Clinicians must maintain oversight and ensure AI recommendations align with best practices, ethical considerations, and individual patient needs. Clear communication with patients about how AI/ML is used in their care, the rationale behind recommendations, and potential limitations builds trust and promotes shared decision-making.
